# Safety and functional enrichment of gut microbiome in healthy subjects consuming a multi-strain fermented milk product: a randomised controlled trial

**DOI:** 10.1038/s41598-020-72161-w

**Published:** 2020-09-29

**Authors:** Anne-Sophie Alvarez, Julien Tap, Isabelle Chambaud, Stéphanie Cools-Portier, Laurent Quinquis, Pierre Bourlioux, Philippe Marteau, Eric Guillemard, Juergen Schrezenmeir, Muriel Derrien

**Affiliations:** 1 Danone Nutricia Research, Palaiseau, France; 2 Faculty of Pharmacy - Paris-Sud University, Chatenay-Malabry, France; 3 APHP, Sorbonne Université - INSERM-ERL 1157 - UMR7203, Hôpital Tenon, Paris, France; 4 Clinical Research Center Kiel, Kiel Innovation and Technology Center, Schauenburgerstr., Kiel, Germany

**Keywords:** Ecology, Microbiology, Gastroenterology

## Abstract

Many clinical studies have evaluated the effect of probiotics, but only a few have assessed their dose effects on gut microbiota and host. We conducted a randomized, double-blind, controlled intervention clinical trial to assess the safety (primary endpoint) of and gut microbiota response (secondary endpoint) to the daily ingestion for 4 weeks of two doses (1 or 3 bottles/day) of a fermented milk product (Test) in 96 healthy adults. The Test product is a multi-strain fermented milk product, combining yogurt strains and probiotic candidate strains *Lactobacillus paracasei* subsp. *paracasei* CNCM I-1518 and CNCM I-3689 and *Lactobacillus rhamnosus* CNCM I-3690. We assessed the safety of the Test product on the following parameters: adverse events, vital signs, hematological and metabolic profile, hepatic, kidney or thyroid function, inflammatory markers, bowel habits and digestive symptoms. We explored the longitudinal gut microbiota response to product consumption and dose, by 16S rRNA gene sequencing and functional contribution by shotgun metagenomics. Safety results did not show any significant difference between the Test and Control products whatever the parameters assessed, at the two doses ingested daily over a 4-week-period. Probiotic candidate strains were detected only during consumption period, and at a significantly higher level for the three strains in subjects who consumed 3 products bottles/day. The global structure of the gut microbiota as assessed by alpha and beta-diversity, was not altered by consumption of the product for four weeks. A zero-inflated beta regression model with random effects (ZIBR) identified a few bacterial genera with differential responses to test product consumption dose compared to control. Shotgun metagenomics analysis revealed a functional contribution to the gut microbiome of probiotic candidates.

## Introduction

Probiotics are defined as live microorganisms that confer a health benefit to the host when administered in adequate amounts^1,2^. Diverse potential health effects of probiotics have been studied in humans, and the evidence of efficacy is strongest for the prevention of necrotizing enterocolitis (NEC), acute respiratory tract infections and antibiotic-associated diarrhea, and for the treatment of acute infectious diarrhea, especially pediatric, and infant colic^3,4^. Probiotics effects were generally both disease- and strain-specific but meta-analyses performed on studies using different strains also provided some evidence that certain effects are shared among different strains^5^. The health benefits of probiotics are thought to be driven by diverse mechanisms, including modulation of the immune response, supporting barrier integrity, and influencing the gut microbiota. Probiotics may interact with resident communities directly, through trophic interactions, or indirectly, by altering the production of host-derived molecules^6–8^. Many studies have investigated the response of the gut microbiota to probiotic consumption, mostly based on 16S rRNA gene amplicon sequencing (reviewed by^8,9^). Shotgun metagenomics-based methods have revealed changes in the metabolism of plant polysaccharides and SCFA production suggestive of an expansion of the carbohydrate-metabolizing capacity of the microbiota during the transient colonization of the gastrointestinaI tract by the ingested strains^10–14^.

A product containing yogurt and three probiotic candidate strains, *Lactobacillus paracasei* CNCM I-3689, *Lactobacillus rhamnosus* CNCM I-3690 and *Lactobacillus paracasei* CNCM I-1518, combined in a fermented milk matrix, was designed. Several preclinical studies have suggested that these three strains could modulate the gut barrier and/or the gut microbiota^15–19^. *L. paracasei* CNCM I-3689 decreased the translocation and dissemination of *Listeria monocytogenes*^15^, induced the clearance of vancomycin-resistant enterococci^16^ and promoted the resilience of some members of the microbiota following exposure to an antibiotic challenge in mice^16^. In addition, *L. rhamnosus* CNCM I-3690 counteracted the increase in intestinal permeability induced by mild inflammation^18,19^, and prevented blooms of the pathobiont *Bilophila wadsworthia* and related deleterious host metabolic effects in mice fed with a high-fat diet^17^. Moreover, *L. paracasei* CNCM I-1518, modulated the activity of *Faecalibacterium prausnitzii* in an in vitro gut model^20^. This strain was extensively studied in clinical trials, in the form of a fermented milk product that had beneficial effects on the incidence and duration of common respiratory and gastrointestinal infections, immunomodulation and antibiotic-associated-diarrhea, and this product was well-tolerated in various populations, including children, adults and the elderly^21–27^. The consumption of the product containing *L. paracasei* CNCM I-1518 and CNCM I-3689 and *L. rhamnosus*, CNCM I-3690 strains decreased *Citrobacter rodentium*-induced colonic crypt hyperplasia and prevented the loss of some bacterial genera in mice^28^. We, therefore, hypothesized that a fermented milk containing these three strains would have beneficial effects on digestive health in humans.

Systematic reviews investigating the safety of probiotics have concluded that their use in humans does not lead to an increase in the risk of adverse events^29,30^. Since these meta-analyses, several additional phase 1 safety studies have been conducted, also documenting safety of certain probiotics used in different population groups^31–34^. However, safety has not been assessed thoroughly in many studies^29,30^, and some vulnerable patients in specific condition have also been identified at higher risk for adverse events in case of probiotic consumption^29^. Further, few studies have assessed the effect of the dose on probiotic safety^35–37^. *L. paracasei* and *L. rhamnosus* have “qualified presumption of safety” status as notified by the European Food Safety Authority^38^, but additional safety evaluations, including assessment of transmissible antibiotic resistance genes, must be conducted prior to use of a QPS strain in food^1^.

In this study, as a primary aim we assessed the safety in healthy human volunteers, of the daily ingestion of two different doses (1 or 3 bottles/day) of a fermented milk product including yogurt starters supplemented with *L. paracasei* CNCM I-1518, *L. paracasei* CNCM I-3689 and *L. rhamnosus* CNCM I-3690. As a secondary aim, we then explored the longitudinal and dose response of the gut microbiota to this product by 16S rRNA gene sequencing. Finally, we assessed the functional contribution of the three probiotic candidates to gut microbiome function by shotgun metagenomic sequencing.

## Materials and methods

### Study design

**Figure 1:**
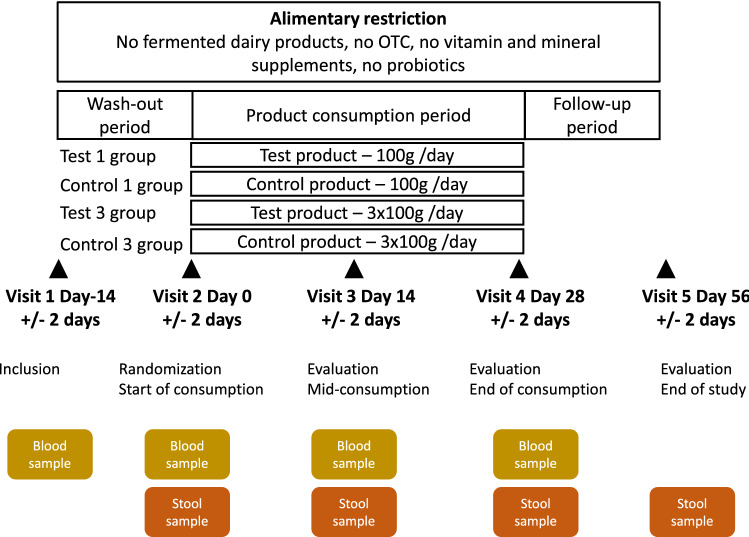
Clinical study design.

The study was a single-center, randomized, double-blind, controlled study, stratified by sex in four parallel groups with a 1:1:1:1 allocation ratio: the Test 1, Control 1, Test 3 and Control 3 groups, receiving one (Test 1 and Control 1) or three (Test 3 and Control 3) bottles per day of the Test or the Control product. The study period was split into three subperiods (Fig. 1): a 2-week washout period (day 14 to day 0), a 4-week period of Test or Control product consumption (day 0 to day 28) and a 4-week follow-up period (day 28 to day 56). Dietary restrictions were imposed throughout the entire study period (from day 14 to day 56), with prohibition of the consumption of other fermented dairy products, probiotics, vitamins and mineral supplements, to limit potential interference with the evaluation of the Test product effects. Each subject attended five visits to a clinical unit (Harrison Clinical Research, Munich, Germany): inclusion visit (V1-day 14), randomization visit (V2-day 0), two evaluation visits (V3-day 14, V4-day 28), and an end-of-study evaluation visit (V5-day 56). Blood and stool samples were collected for assessments of eligibility and of the safety evaluation criteria at V1, 2, 3 and 4 (blood) and V2, 3, 4, and 5 (stool). Each visit had to take place within 2 days of the scheduled visit date (± 2 days) to ensure a consistent adequacy between the times of clinical and biological measures and the duration of each corresponding period of product intake or follow-up between subjects. This study was performed in accordance with the principles of the Declaration of Helsinki, the French Huriet law, and ICH-GCP recommendations, and was approved by the ethics committee of the Bavarian Medical Association, Munich, Germany. All volunteers provided written informed consent. This trial was registered on the ClinicalTrials.gov, with the registration number NCT01108419 (date of registration April 22, 2010). The study was funded by Danone Research (France).

### Subject selection

Subjects were screened between March and April 2010, and the study lasted from March 29th 2010 (first subject included) to June 25th 2010 (last subject completed). The following eligibility criteria were assessed at subject inclusion (V1). The inclusion criteria were: male or female volunteers providing written informed consent, aged from 18 to 55 years, with a body mass index (BMI) of 18.5 to 30.0 kg/m^2^, free-living and considered to be in good health on the basis of a clinical examination, with a normal defecation pattern and either menopausal or with an approved method of contraception if female. Non-inclusion criteria were: any allergy, hypersensitivity to any component of the study product, including lactose, systemic or topical treatment (at the time of inclusion or in the previous 4 weeks) likely to interfere with the evaluation of the study parameters (antibiotics, intestinal or respiratory antiseptics, antirheumatic agents, anti-inflammatory drugs [except for aspirin or equivalent at doses preventing from platelet aggregation or blood clotting] and steroids prescribed for chronic inflammatory diseases), any symptoms of respiratory or gastrointestinal common infectious diseases, a history of chronic metabolic or gastrointestinal disease, abdominal pain or any other severe progressive or chronic disease (cardiac, respiratory, etc.), immunodeficiency, eating disorders or a medicated diet, pregnancy or breast-feeding. The following eligibility criteria were also assessed at the randomization visit (V2): compliance with the dietary and medication restriction (as defined in the non-inclusion criteria) between V1 and V2, negative pregnancy test and parameters within the normal range in the blood samples collected at V1, and absence of common infectious disease symptoms.

### Product intervention

The Test product was a fermented dairy drink containing *Lactobacillus paracasei* CNCM I-1518, *Lactobacillus paracasei* CNCM I-3689 and *Lactobacillus rhamnosus* CNCM I-3690 strains, with 10^7^ to 10^9^ colony-forming units (CFU)/g of product, and four yogurt strains (*Lactobacillus bulgaricus* CNCM I-2787*, Streptococcus thermophilus* CNCM I-2773, *Streptococcus thermophilus* CNCM I-2835, *Streptococcus thermophilus* CNCM I-2778). Counts were measured for each of the bacterial strains present in the Test product, at the start and end of the authorized storage period (shelf life). Means and ranges of strains counts from the batches of product used in the study are provided in Supplementary Table S1. The Control product was a non-fermented dairy drink, acidified with lactic acid and containing pectin as a stabilizer. Both the Test and Control products were sweetened and multi-fruit flavored. Both products were similar in terms of their appearance, packaging, nutritional content (isocaloric) and taste, to ensure the maintenance of double-blinding (both the participants and key study personnel, including the outcome assessors) until the database was locked and the request by the statistician for unblinding (the only staff not blinded being those involved in the preparation of the study products). Products were manufactured in a pilot plant approved by the national health authorities for the production of dairy products for human consumption. They were supplied by Danone Research, France and stored at + 4 ± 2 °C, with a shelf life of 37 days. Analyses were performed to guarantee the absence of microbiological contaminants in all products. Subjects were randomly assigned to the Test or Control group according to a randomization list established before the start of the study by an external statistician. The randomization list contained balanced blocks, stratified by sex, with the allocation of an incremental number linked to product number given by an IWRS system, and was kept confidential at the sponsor’s premises in order to ensure allocation concealment. The subjects were then asked to ingest either one (100 g) or three (3 × 100 g) bottles of the Test or Control product daily, in accordance with their randomization group, for the entire 4-week product-consumption period (28 days). Subjects with three doses per day were recommended to consume no more than two doses at the same time. Compliance was evaluated by the investigator on the basis of the daily reporting of product consumption by each participant in a personal diary and a count of unused bottles.

### Outcomes

The primary aim of the study was to compare product safety between the Test 1 and Control 1 groups over the 4-week period of product consumption. The safety evaluation was based on the following parameters: adverse events, physical examination, hematology, metabolism profile, markers of hepatic, kidney and thyroid function, inflammatory markers, bowel habits and frequency of digestive symptoms. Additional information about safety parameters is provided in Supporting Information.

As secondary criteria, safety parameters were also analyzed for the Test 3 and Control 3 groups, over the period of product consumption (V2 to V4), and for both 1 and 3 product doses during other periods: the follow-up period (V4–V5) and the whole experimental period (V2–V5). Stool samples were also subjected to testing to detect and quantify the strains present in the Test product and to analyze the microbiota, for both doses and different study periods (see details and methods below).

### Procedure

At each visit, from V1 to V5, subjects underwent a physical examination and vital signs were recorded. Subjects completed a personal diary throughout the 10-week study period, which was collected and examined at each visit by the investigator. This diary included daily reports of study product consumption, the intake of unauthorized products, concomitant medication, symptoms, frequency and consistency of stool and a weekly scoring from the Frequency of Digestive Symptoms questionnaire. The physical activity and smoking habits of the subjects were recorded at each visit. Blood samples were collected for analyses after overnight fasting every two weeks from V1 to V4. The measure of calprotectin concentration, the detection and quantification of strains from the Test product, and the evaluation of the microbiota profile were performed on stool samples collected at each visit from V2 to V5. The study was performed in accordance with the protocol and the statistical analysis plan with no major change during the course of the trial.

### Safety monitoring committee

A safety and monitoring committee (SMC), composed of three independent experts in internal medicine, hepato-gastro-enterology and pharmacology, performed an unblinded review of the subject withdrawals, the protocol deviations, the statistical analyses of study parameters and the individual data in the event of abnormal values for safety results. The statistical results were presented after the database lock by the study scientist and statistician to the SMC during two meetings. The SMC then presented its conclusions concerning the safety of the daily ingestion of the Test product at the two doses evaluated.

### Stool collection, DNA extraction

We collected fecal samples from 90 subjects at four time points (Test 1 (N = 22), Test 3 (N = 23), Control 1 (N = 21), Control 3 (N = 24)) in RNAlater solution (Ambion, Courtaboeuf, France). Fecal DNA was extracted by mechanical lysis (FastprepFP120; ThermoSavant, Illkirch, France) followed by phenol/chloroform-based extraction, as previously described^39^. The DNA preparation was subjected to quality control by spectrophotometry on a NanoDrop 2000c spectrophotometer (Thermo Fisher). The DNA was analyzed by quantitative polymerase chain reaction (qPCR), 16S rRNA gene sequencing and whole-genome sequencing.

### Quantitative PCR

Three strains, *Lactobacillus paracasei subsp. paracasei* CNCM I-1518, *Lactobacillus paracasei subsp. paracasei* CNCM I-3689 and *Lactobacillus rhamnosus* CNCM I-3690, were quantified by qPCR, as previously described^39^, with specific primers (Supplementary Table S2). Values were reported as median and interquartile range.

### 16S RNA gene sequencing, processing and analysis

16S RNA gene sequencing was performed as previously described^18^. Amplification was performed with the V3-V4 primers for the 16S rRNA (forward: CCTACGGGNGGCWGCAG, reverse: GACTACHVGGGTATCTAATCC). The samples were loaded into flow cells in an Illumina MiSeq 300PE Sequencing Platform, in accordance with the manufacturer’s instructions. Analyses were performed with QIIME (v. 19). The sequences were filtered for quality and a mean of 99,437 ± 36,973 reads per sample were retained. Reads were clustered into operational taxonomic units (OTUs; 97% identity threshold) with VSEARCH, and representative sequences for each OTU were aligned and taxonomically assigned with the SILVA database (v. 119). Alpha-diversity was assessed at genus level. Beta diversity was assessed with Bray–Curtis dissimilarity, Jensen-Shannon divergence, and weighted and unweighted UniFrac on genera and OTUs.

### Metagenomic shotgun sequencing and preprocessing

Following standard DNA quality control and quantification, sequencing libraries were prepared with the Nextera XT DNA sample preparation kit in accordance with the manufacturer's instructions. An overview of the bioinformatic pipeline used in this study is provided in Supplementary Fig. S1. We generated a mean of 35 million (± 8 million) paired-end reads per sample. Read cleaning, filtering and mapping were performed with NGLess version 0.7^40^. An augmented catalog was built from the Integrated Gene Catalog (IGC)^41^ enriched with genes from the sequencing and de novo assembly of these 107 metagenomes and the seven bacterial genomes present in the Test product (Supplementary Fig. S2). Mapping and count matrix generation were also performed with NGLess. The taxonomic profile was extracted from the count matrix with the Metagenomic Species Pan-Genomes database^42^. For functional characterization, the catalog was annotated with functional data from the Kyoto encyclopedia of genes and genomes (KEGG, https://www.genome.jp/kegg/)^43^.

### Functional contribution

Metagenomic gene count matrices were aggregated at KEGG orthologous (KO) levels, for the whole gene set and for genes from *L. rhamnosus* and *L. paracasei* from the Test product only. We estimated the contribution of the Test product to each KO, by dividing each KO relative abundance level for the Test product by the corresponding value for the whole gene set. A pseudocount of one was added. Corresponding KO relative abundances for the 31 universally distributed marker genes from Ciccarelli et al.^44^ were also obtained, to estimate the minimal functional contribution of each Test product gene. All KOs for the Test product with a contribution strictly higher than the minimal contribution, constituting a significant functional contribution of the Test product to the gut metagenome, were extracted for downstream analysis. KEGG BRITE and module annotations were used to explore this functional contribution, focusing on enzymes and transporters. We then assessed the extent to which this significant functional contribution set was shared by the other metagenomic species pan-genomes (MSPs).

### Statistical analysis

#### Clinical parameters

No data on adverse events were available to assess the sample size required. The decision to include 24 subjects per group was thus made on the basis of previously published safety studies^45,46^. For assessment of the safety of consuming the Test product, in comparison to the Control product, adverse events were recorded (MedDRA version 13) and used to evaluate the number of subjects with at least one adverse event, and the total number of adverse events overall, and by relationship to the study product, intensity, seriousness, action taken, and subject outcome. Additional physical examination data, blood parameters, calprotectin concentration in feces, and questionnaires about bowel movements, stool consistency and the frequency of digestive symptoms were collected throughout the period of product consumption and were analyzed as raw data or in terms of clinical significance relative to the baseline value. No formal statistical tests has been performed to assess the safety and study conclusions were based on nominal statistics as described hereafter, on individual data and on overall agreement of the SMC. For quantitative variables, Cohen’s *d* was calculated for the change from baseline after 4-week product consumption in Test and Control groups as follows: *Cohen’s d* = *(Average raw change from baseline in Test group − Average raw change from baseline in Control group)/Pooled standard deviation at baseline*. Cohen’s *d* values around 0.50 are considered to be of medium magnitude, and those around or above 0.80 are considered to be large^47,48^. In this study, an absolute Cohen’s *d* value above 0.5 was considered to be large enough to detect a potential difference between the Test and Control groups. For qualitative binary parameters, the relative risk (RR) and its 95% confidence interval (CI) were calculated by the normal approximation method. Safety analyses were performed on all randomized subjects who had consumed the Test or Control product at least once, i.e. the full analysis set (FAS) population. Statistical analyses were performed with the Statistical Analysis Systems statistical software package version 9.1.3 (Windows XP Professional; SAS Institute, Cary, NC, USA).

#### Gut microbiota

We used non-parametric tests to analyze qPCR data, alpha and beta-diversity, gene and species richness within individuals, between groups, at baseline and over time. Differential analyses were performed with DESeq2 (version 1.14.1)^49^ and ZIBR^50^. For all tests, the alpha risk was set at 0.05 after FDR adjustment by the Benjamini–Hochberg procedure. Network analysis was performed with the SPIEC-EASI R package (version 1.0.7^51^). All statistical analyses were performed, and graphs were plotted with R software (version 3.6.0). Details of the analyses and parameters are provided in Supporting Information.

## Results

### Subject enrollment, population at baseline and compliance

**Figure 2:**
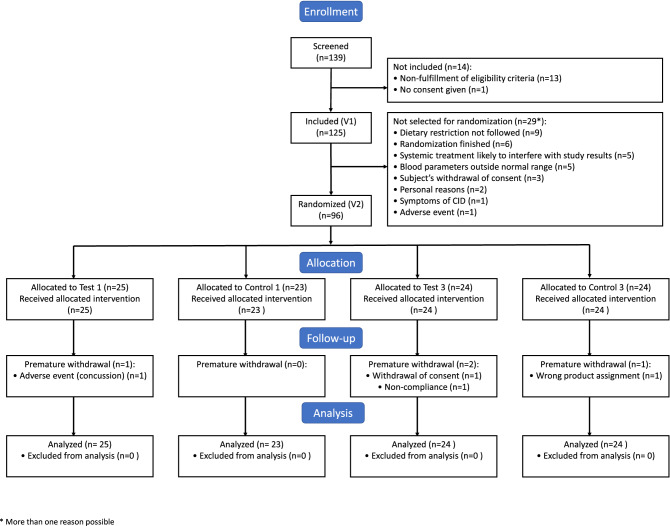
Flowchart for the study population.

Of the 139 subjects screened, 125 subjects were included in the study at V1. Then, 96 subjects (FAS population) were randomized to one of the four groups: 25 to Test 1, 23 to Control 1 and 24 each to the Test 3 and Control 3 groups (Fig. 2). One subject was randomized in the wrong stratum, as a woman rather than a man, explaining the odd number of subjects in the Test and Control 1 groups, but was analyzed as man. Four randomized subjects (4%) withdrew prematurely as they stopped their participation in the study before completion (one in Test 1, two in Test 3, and one in the Control 1 group).

A summary of subject characteristics at baseline is shown in Table 1. Forty-four men and 52 women were randomized. Most subjects were in their early thirties (range 18 to 55 years) with a BMI from 18.6 to 31.1 kg/m^2^. The Test and Control groups were well-balanced at baseline for subject age, sex and BMI (Table 1), and for most of the safety parameters, which were similar between groups, with mean values in the normal range on day 0, for all groups. These include blood parameters, vital signs, calprotectin concentration in feces, frequency of bowel movements and the consistency of the feces (data not shown). A few parameters were not balanced or slightly imbalanced between groups at baseline, including proportion of current smokers, subjects reporting a regular physical activity, medical or a surgical history, concomitant medication (Table 1) and blood hs-CRP (see Supporting Information), but they were considered not to interfere with the product safety evaluation. The study being randomized, these imbalances were due to chance. Additional information on the baseline characteristics of subjects and the not balanced parameters can be found in Supporting Information.

**Table 1:** Subject characteristics at baseline.

	Test 1 (*N* = 25)	Control 1 (*N* = 23)	Test 3 (*N* = 24)	Control 3 (*N* = 24)
Age^a^ (years), median (min–max)	30 (20–52)	32 (18–53)	29 (20–55)	34.5 (25–55)
Male^a^, *n* (%)	11 (44%)	11 (48%)	11 (46%)	11 (46%)
BMI^b^ (kg/m^2^), median (min–max)	24.4 (20.3–29.4)	24.9 (19.6–30.1)	24.6 (21.5–31.1)	22.7 (18.6–28.1)
Smoking status^a^, n (%)				
Never	11 (44%)	17 (74%)	14 (58%)	11 (46%)
Previous	2 (8%)	1 (4%)	3 (13%)	7 (29%)
Current	12 (48%)	5 (22%)	7 (29%)	6 (25%)
Regular physical activity^a^, *n* (%)	11 (44%)	8 (35%)	18 (75%)	13 (54%)
Medical or surgical history^a^, *n* (%)	3 (12%)	6 (26.1%)	5 (20.8%)	3 (12.5%)
Concomitant medication^b^, *n* (%)	11 (44%)	8 (35%)	9 (38%)	9 (38%)

^a^At inclusion (V1).

^b^At randomization (V2).

Subject compliance with product consumption was high, with a percentage [mean (SD)] of product intake (observed number/theoretical number) of 98.6 (8.8)% in the Test 3 group and 99.5 (2.2)% each for the Test 1, Control 1 and Control 3 groups. The median duration of product consumption was 27 days in all four groups (range 13 to 29 days). A small number of unauthorized dietary products (1 to 8 units) were consumed by 28% of the subjects, evenly distributed among groups, during the whole study period. Additional information about study quality, including major deviations and missing data, is provided in Supporting Information.

### Safety evaluation

Some subject-related factors measured at baseline that could affect the safety evaluation were also assessed throughout the whole study duration. Physical activity and smoking habits remained stable from baseline. The number of subjects with concomitant medication differed between Test and Control groups, mostly due to the use of contraceptives and of anti-inflammatory/antirheumatic treatments in only a few subjects. These factors were not, therefore, expected to affect the safety evaluation.

#### Adverse events

The adverse events (AE) recorded are described in Table 2. About half the subjects reported at least one adverse event during the 4-week product-consumption period, with similar frequencies in the Test 1, Test 3 and Control 1 groups (50 to 52%), and a slightly higher percentage in the Control 3 group (67%). The most common AE were gastrointestinal events, mostly flatulence, abnormal borborygmi and abdominal pain. In all four groups, most of the AE were considered to be related to the study product. The risk of experiencing an event related to study product consumption was lower in the Test groups than in the Control groups (1 bottle: RR = 0.84, 95% CI [0.47–1.52]; 3 bottles: RR = 0.77, 95% CI [0.42–1.40]). The risk of experiencing a gastrointestinal event was similar in the Test 1 and Control 1 groups and lower in the Test 3 than in the Control 3 group (RR = 0.67, 95% CI [0.38–1.17]). During the follow-up period, a larger number of subjects in the Test 1 group than in the Control 1 group reported at least one AE or one gastrointestinal AE, but the numbers of AE related to the study product were low and similar in the two groups (Table 2). For the same period, the total number of AE and the number of gastrointestinal AE were lower in the Test 3 than in the Control 3 group and the numbers of AE related to the study product were identical in these two groups. AE related to a clinically significant level of calprotectin in the feces were reported by three subjects in the Test 1 group and one in the Control 1 group, but the opposite pattern was observed for the higher dose, with no subjects in the Test 3 group and three in the Control 3 group reported such AE. All the AE observed throughout the entire study period were mild to moderate in intensity, with the exception of two AE of severe intensity reported by two subjects in the Test 1 group (1 case of concussion, 1 high fecal calprotectin concentration), both of which were considered unlikely to be related to the product. Only one non-product related serious adverse event (an abscess) was reported in the Control 3 group. No specific action was taken after the occurrence of an AE, with the exception of the withdrawal from the study of the subject with concussion.

**Table 2:** Most common adverse events.

	Test 1 (*N* = 25)	Control 1 (*N* = 23)	RR (95% CI)	Test 3 (*N* = 24)	Control 3 (*N* = 24)	RR (95% CI)
**Product consumption**						
AE [*n* (%)]	13 (52%)	12 (52%)	1.00 (0.58–1.72)	12 (50%)	16 (67%)	0.75 (0.46–1.22)
Serious AE [*n* (%)]	0	0	NA	0	1 (4%)	NA
AE of severe intensity [*n* (%)]	1 (4%)	0	NA	0	0	NA
AE related to the study product [*n* (%)]^a^	11 (44%)	12 (52%)	0.84 (0.47–1.52)	10 (42%)	13 (54%)	0.77 (0.42–1.40)
Gastrointestinal AE [*n* (%)]	11 (44%)	10 (43%)	1.01 (0.53–1.92)	10 (42%)	15 (63%)	0.67 (0.38–1.17)
Flatulence	10 (40%)	9 (39%)	1.02 (0.51–2.06)	9 (38%)	14 (58%)	0.64 (0.35–1.19)
Abnormal borborygmi	4 (16%)	4 (17%)	0.92 (0.26–3.26)	6 (25%)	7 (29%)	0.86 (0.34–2.18)
Abdominal pain	2 (8%)	4 (17%)	0.46 (0.09–2.28)	2 (8%)	1 (4%)	2.00 (0.19–20.61)
Pain^b^	0	0	NA	2 (8%)	0	NA
Nasopharyngitis	2 (8%)	1 (4%)	1.84 (0.18–18.96)	1 (4%)	1 (4%)	1.00 (0.07–15.08)
Fecal calprotectin concentration^c^	0	3 (13%)	NA	0	0	NA
Headache	0	0	NA	1 (4%)	3 (13%)	0.33 (0.04–2.98)
**Follow-up**						
AE [*n* (%)]	14 (56%)	8 (35%)	1.61 (0.83–3.11)	7 (29%)	10 (42%)	0.70 (0.32–1.53)
Serious AE [*n* (%)]	0	0	NA	0	0	NA
AE of severe intensity [*n* (%)]	1 (4.0%)	0	NA	0	0	NA
AE related to the study product [*n* (%)]^a^	2 (8%)	3 (13%)	0.61 (0.11–3.35)	2 (8%)	2 (8%)	1.00 (0.15–6.53)
Gastrointestinal AE [*n* (%)]	12 (48%)	7 (30%)	1.58 (0.75–3.31)	6 (25%)	9 (38%)	0.67 (0.09–1.94)
Flatulence	9 (36%)	7 (30%)	1.18 (0.53–2.66)	5 (21%)	8 (33%)	0.63 (0.24–1.64)
Abnormal borborygmi	5 (20%)	3 (13%)	1.53 (0.41–5.71)	1 (4%)	1 (4%)	1.00 (0.07–15.08)
Abdominal pain	4 (16%)	2 (9%)	1.84 (0.37–9.12)	1 (4%)	2 (8%)	0.50 (0.05–5.15)
Diarrhea	0	1 (4%)	NA	2 (8%)	0	NA
Fecal calprotectin concentration^c^	3 (12%)	1 (4%)	2.76 (0.31–24.7)	0	3 (13%)	NA

In number (n) and percentage of subjects with at least one AE. Occurrence of AE by type is detailed for AE observed in at least in 2 subjects in one group.

^a^Possibly, probably or highly probably.

^b^Related to general disorders and administration site conditions.

^c^Subjects with AE relating to an increase of calprotectin concentration from < 50 μg/g or from 50 to 100 μg/g at baseline, corresponding to excluded and possible inflammatory gastric disease respectively, to a concentration > 100 μg/g during the study, corresponding to a confirmed inflammation.

#### Bowel movements, digestive symptoms and vital signs and biological parameters

Details on additional data on safety parameters including bowel movements, digestive symptoms, vital signs and biological parameters in blood and feces samples can be found in Supporting Information. Briefly, during the 4-week product consumption or the follow-up periods, no clinically significant changes in each group and no difference between groups was observed for all the following parameters: defecation frequency, stool consistency scores, composite score and frequency of digestive symptoms (abdominal pain, bloating, flatulence and rumbling) or vital signs. For biological parameters, clinically relevant changes were minimal and rarely observed and were equivalent after 4 weeks of product consumption for all parameters in all groups.

### Test product strains are detected transiently in the gut microbiota

**Figure 3:**
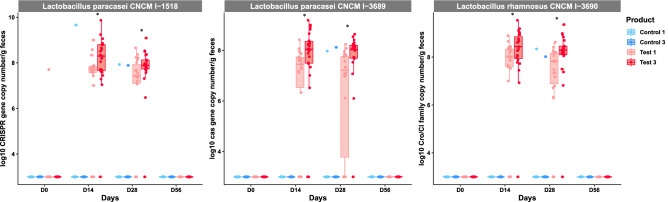
Detection of strains in fecal samples. Quantification of three probiotic candidates by qPCR with strain-specific primers on fecal samples before (D0), during (D14 and D28) and after (D56) the period of Test product consumption. Data are expressed as Log10 gene copy number/g feces. *p < 0.05, Mann–Whitney test for the comparison between 1 daily dose (Test 1) and 3 daily doses (Test 3) of product.

*L. paracasei* CNCM I-1518, *L. paracasei* CNCM I-3689 and *L. rhamnosus* CNCM I-3690 strains were quantified by strain-specific qPCR (Fig. 3). None of these three strains were detected at baseline, but the levels of all three strains increased during the period of consumption, subsequently decreasing to levels below the threshold of detection 28 days after the cessation of product consumption. After four weeks of consumption *L. paracasei* CNCM I-1518 was detected at a median [Q1–Q3] of 7.43 [7.10–7.93] and 7.89 [7.73–8.13] log10 gene copy number/g of feces in Test 1 and Test 3 respectively. Similarly, *L. paracasei* CNCM I-3689 was detected at 7.21 [3.77–7.75] and 8.03 [7.69–8.2] log10 gene copy number/g of feces in Test 1 and Test 3 respectively. *L. rhamnosus* CNCM I-3690 was detected at 7.82 [6.88–8.17] and 8.29 [8.1–8.46] in Test 1 and Test 3 respectively (Fig. 3). *L. paracasei* CNCM I-1518, CNCM I-3689 and *L. rhamnosus* CNCM I-3690 were therefore transiently detected in the gut microbiota of healthy adults after consumption of the Test product. Higher levels of all three strains were detected in subjects who consumed three products/day, in tests performed on D14 and D28 (Mann–Whitney test, *p* < 0.05, FDR).

### Differential analysis reveals a modest response of the gut microbiota to the test product

**Figure 4:**
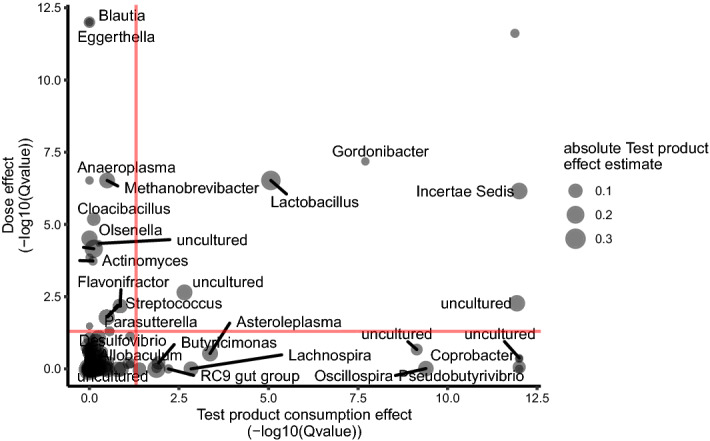
Genera with differential abundances during the study identified by ZIBR. Effect of consumption as a function of dose, for each genus, modeled with ZIBR. The reported values are *p* values corrected for multiple testing (FDR).

We compared the dynamics of the gut microbiota response to the intervention between groups, by 16S rRNA gene sequencing (Test1, Test 3, Control 1, Control 3). We first investigated whether global microbiota structure differed between groups or doses. No significant difference in either alpha nor beta-diversity was found between groups or between doses, at any time point, for any of the metrics assessed, suggesting that the intervention did not elicit significant global changes in microbiota structure (Supplementary Fig. S3, *p* > 0.05, Kruskal–Wallis). DESeq2 analysis found no differential abundance between groups or doses, for any genus, during the study (FDR adj. *p* < 0.05, DESeq2-based Wald test). We then used (ZIBR), a dedicated approach specifically designed for zero-inflated datasets that can handle repeated measurements, to identify bacterial genera displaying transiently different responses to the two doses of Test product versus Control product. This approach was based on the hypothesis that transiently higher levels of probiotic candidates, as observed (Fig. 3), would induce transient differences in the abundance of other bacterial genera. ZIBR analysis showed that some genera responded either only during the Test product consumption period (*Pseudobutyrivibrio*, *Coprobacter*, *Oscillospira*) or only to a Test product-dose effect (*Blautia*, *Methanobrevibacter*, *Eggerthella*). Six genera were both dose- and consumption period-responsive (Fig. 4), suggesting that they differed in abundance between doses during consumption of the Test product. The genera included *Holdemania*, *Gordonibacter, Lactobacillus,* an unclassified Mollicutes (RF-9) and two unclassified genera from Clostridiales. We then used a multivariate visualization to confirm the ZIBR results. Principal component analysis (PCA) on center log-ratio (Clr)-transformed data for the six bacterial genera differentiated between subjects from the Test 3 and Control 3 groups at D14 and D28, based on PC2 (accounting for 19.39% of variation), (Mann–Whitney *p* = 0.03 FDR) (Supplementary Fig. S4). We, therefore, performed a metagenomic analysis on this subset of subjects (D0 and D28). An augmented catalog was first built from the IGC^41^ enriched with the genes from the de novo assembly of metagenomes in this study and from the bacterial genomes of the strains present in the Test product. DESeq2 analysis identified no Metagenomic Species Pan-genomes (MSPs) or gut modules differing in abundance between the Test 3 and Control 3 groups after four weeks of consumption (FDR adj. *p* > 0.05, DESeq2-based Wald test). These results suggest that Test product consumption does not alter the global structure or function of the gut microbiota but shows that a few genera respond to the intervention or its dose.

### Functional contribution and covariation of test product strains within the gut microbiome

**Figure 5:**
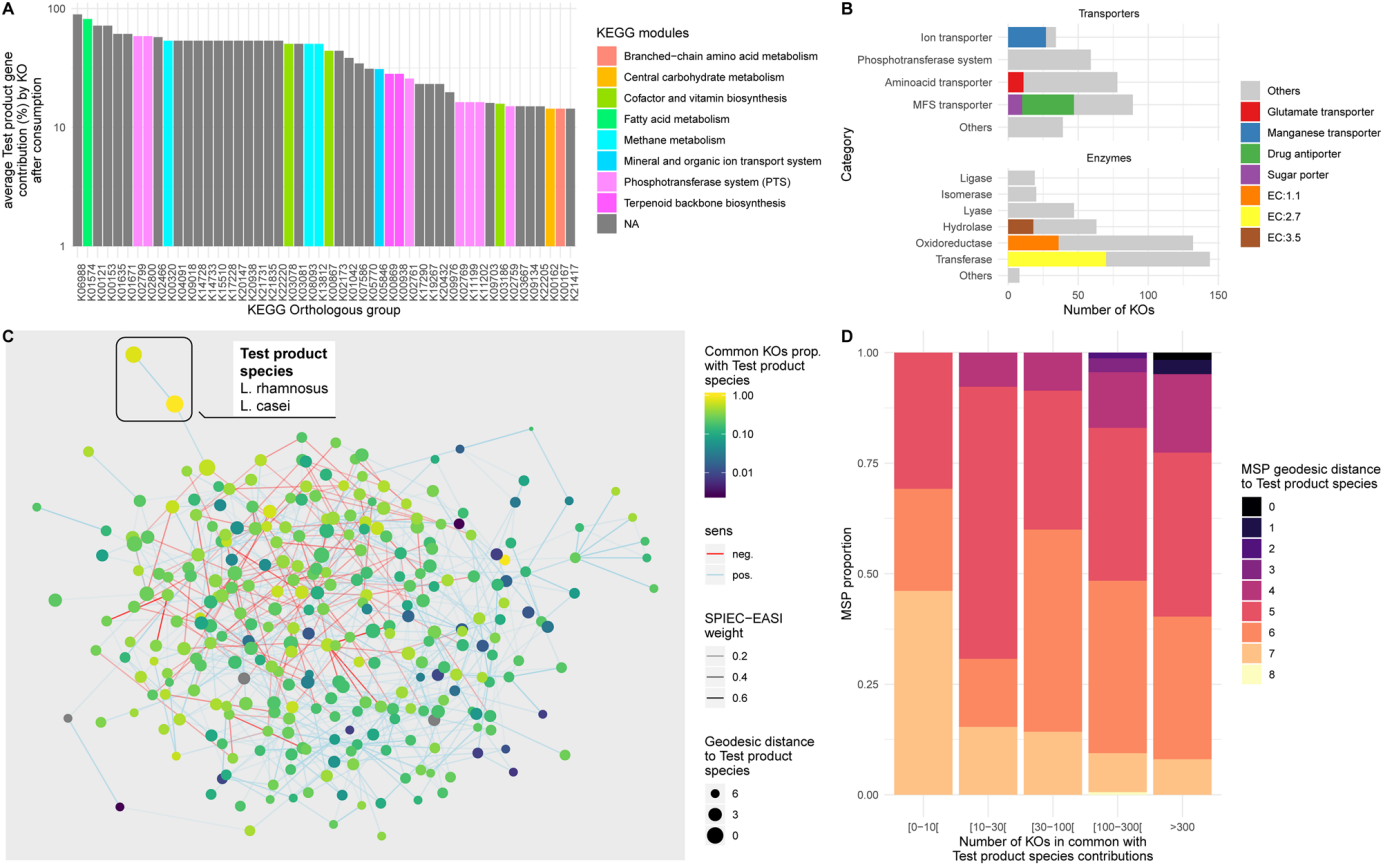
Functional contribution of Test product strains to the gut microbiome and their association with resident species. (**A**) Ranked barplot of 50 of 798 KOs with the highest relative abundance contributions. NA corresponds to unclassified KOs. (**B**) Ranked barplot of the distribution of the 798 KOs within transporter and enzyme KEGG BRITE category. The colors indicate the most dominant functions. (**C**) Microbial co-abundance network based on the SPIEC-EASI method. Each dot represents a single MSPs. Positively and negatively co-abundant MSPs are connected by blue and red lines, respectively, the thickness of which is determined by weight in the SPIEC-EASI, model. Node colors indicate the number of shared specific functions from a list of 798 KOs contributed by the Test product probiotic candidate species (*Lactobacillus rhamnosus* and *Lactobacillus paracasei*) to the gut microbiota. Node diameter indicates the geodesic edge distance with MSPs. D. Barplot of the number of shared contributive KOs between Test product strains and dominant species as a function of geodesic distance extracted from the abundance co-variation network.

We used shotgun metagenomics to assess the extent of the functional contribution of Test product strains within the microbiome. We focused on the three probiotic candidates, as yogurt strains were previously shown not to survive in an in vitro Gastro-Intestinal Digestive Simulator^52^. 5,452 non-redundant genes (95% similarity) were identified for the three strains, corresponding to 2,176 bacterial (KEGG) ortholog groups (KOs). For each KO, counts for all genes were summed to generate an aggregate estimated count. We assessed the relative contribution of each KO carried by the three probiotic candidates as a proportion of the total originating from all gut microbiome-resident MSPs. 798 KOs were identified (referred to hereafter as “Test product contributive KO”) from the three probiotic candidates (Supplementary Fig. S5, Table S3). Most of these KOs belonged to unassigned KEGG modules, but those that were assigned belonged to the PTS system, branched-chain fatty acids, vitamin, and amino acid modules, making a contribution of up to 90% (Fig. 5A). A more detailed analysis of these KO with KEGG BRITE suggested that they corresponded to transporters (MFS, PTS and ion transporters) and enzymes (oxidoreductase, transferases and hydrolases) (Fig. 5B).

Finally, we explored the covariation of the Test product species (*L. paracasei*, and *L. rhamnosus*) with the resident species of the gut microbiota, by SPIEC-EASI (Fig. 5C). For each gut microbiome-resident MSPs, we assessed the number of KOs detected from the Test product contributive KOs (Fig. 5D). We found that resident MSPs separated from Test product species (mostly *S. thermophilus*) by a smaller number of edges (i.e. more directly connected) shared larger number of KOs through interactions with Test product species than the others (Spearman’s rho =  − 0.12, *p* = 0.047). These results remained valid if only positive interactions were taken into account (Spearman’s rho =  − 0.12 *p* = 0.045), but not if only negative interactions were considered (Spearman’s rho = 0.1 *p* = 0.22). Our findings suggest that MSPs from the Test product co-occur with resident gut microbiome species with which they share a larger number of KOs, probably corresponding to species with a similar function.

## Discussion

In this study, we evaluated the safety of the daily consumption of two doses (1 or 3 bottles/day) of a fermented milk product (Test product) containing a mix of three *Lactobacillus* strains, *L. paracasei* CNCM I-1518*, L paracasei* CNCM I-3689 and *L. rhamnosus* CNCM I-3690*,* selected for their probiotic potential, and four common yogurt strains. Statistical analyses and analyses of individual subjects’ results were performed for adverse events and biological parameters. The results do not raise any safety concerns for the ingestion of the Test product once to three times per day, corresponding respectively to a minimum of 1 × 10^9^ to 3 × 10^9^ and a maximum of 1 × 10^11^ to 3 × 10^11^ CFU/subject/day, according to the range of bacterial count in the product, for each of the three probiotic candidate strains. These results are consistent with previous studies showing that the consumption of other probiotics at a dose of 10^8^ to 10^11^ CFU/day in healthy adult subjects had no significant effect on blood chemistry, metabolic and immune parameters, bowel habits, vital signs or adverse event occurrence, since all these parameters were similar for the placebo products used^45,53,54^.

Then, we sought to explore whether there was a response of gut microbiota to the Test product and according to the dose. First, we tracked the probiotic candidates in fecal samples throughout the study. Using qPCR, we were able to detect all probiotic candidates during the product-consumption period, but none were detected 28 days after the cessation of product ingestion. The transient detection of these strains in feces is consistent with the findings of other studies, most of which have shown that the ingested bacterial strains can be detected for a few days after product ingestion has ceased, but rarely for more than one week^11,55–58^. Some strains may persist for up to few months in healthy adults after their ingestion has ceased. For example, *Bifidobacterium longum* AH1206 was detected in one-third of subjects with a gut microbiota presenting deficiencies of carbohydrate metabolism, and of bacteria related to the ingested strain^12^. We then monitored the dynamics of the gut microbiota response following product consumption, by both 16S rRNA gene sequencing and shotgun metagenomics. We found that gut microbiota structure was modestly modified after product consumption. While DESeq2 detected no differences, a complementary analysis dedicated to longitudinal data from zero-inflated datasets, ZIBR^50^, identified a few genera displaying transient differential modulation between the two doses of the Test product. Genera related to *Lactobacillus*, *Holdemania* and *Clostridiales* were found to respond differently to dose. To our knowledge, this is the first clinical study to evaluate the dose-dependent response of the gut microbiota to a multi-strain product by 16S rRNA gene sequencing. The modest alterations observed after probiotic consumption are consistent with previous studies conducted in healthy adult subjects^11,14,59^, and might be greater in subjects exposed to a challenge^60^ or in younger populations^61^. We explored the functional contribution of the probiotic candidates, which are more likely than the yogurt strains to reach the colon, by analyzing individual KEGG ortholog groups (KOs). We observed that the probiotic candidates had a variable potential to contribute to gut microbiome functions, up to 90% for some KOs. None of the KOs from the Test product strains had a contribution of 100%, suggesting that strains did not provide the gut microbiome with an additional function. Most of the KOs contributing to microbiome function belonged to the phages, vitamin, amino acids, and sugar transport categories. These findings complement those of the study by Maldonado et al. reporting that the administration of *B. longum* AH1206 did not alter the composition of the gut microbiota, but enriched the microbiome in functional genes related to *B. longum*^12^. We also showed that the covariation of Test product species with resident species was associated with the proportion of shared functions. This association was driven by positive interactions, suggestive of cooperation rather than competition for nutrients. A study based on in silico metabolic network models for 154 gut microbes found that species tended to co-occur more frequently with species with which they were in strong competition, across individuals^62^. Our findings suggest strains ingested may enrich some functions of the gut microbiome, and potentially interact with other resident species through the sharing the same metabolic requirements.

The limitations of this study include the small number of subjects, making it impossible to detect rare adverse events. The inclusion of subjects with abnormal values at baseline for some biological parameters may also have limited the evaluation of a potential product effect on these parameters. Furthermore, dietary habits were not assessed and may be a confounding factor with potential effects on parameters such as blood metabolic markers or microbiota profile, although randomization and double-blinding typically equalize such factors between the groups. It is also not possible to establish the respective contribution of each strain of the Test product in the observed effects which can be only considered to be borne by the mix of ferments and their metabolites. However, yogurt strains cannot survive to upper GI tract conditions as shown in a semi-dynamic in vitro model^52^, whereas *L. casei* CNCM-1518 survival in the gut was previously reported in human after consumption in a fermented milk^63^. Also, the three probiotics candidates are more likely major contributors considering their respective effects as demonstrated in former studies as previously described.

In conclusion, the study results suggest that daily consumption (1 or 3 bottles) for four weeks of a fermented milk product containing *L. paracasei* CNCM I-3689, *L. rhamnosus* CNCM I-3690 and *L. paracasei* CNCM I-1518, and yogurt starters, is safe and elicits a structural response of the gut microbiota, although modest, which possibly results from a metabolic activity of probiotic candidates. This might suggest that those transient microbes contribute to the overall gut microbiome metabolism. How those changes may relate to potential health effects remain to be further investigated. Overall, our findings provide data that the transient presence of these strains may enrich some functions of the gut microbiome. This work could provide a basis for the selection of future probiotics to enrich and/or complement microbiome functions.

## Supplementary information

Supplementary Information

Supplementary Figure S1.

Supplementary Figure S2.

Supplementary Figure S3.

Supplementary Figure S4.

Supplementary Figure S5.

Supplementary Table S1.

Supplementary Table S2.

Supplementary Table S3.

Supplementary Table S4.

## Data Availability

The sequence data for the project are publicly available through the European Nucleotide Archive (https://www.ebi.ac.uk/ena/) under accession number PRJEB35769. The source codes used in this study are available from GitHub (github.com/danone/multistrain.tolerance). Strains are available at the following collection https://research.pasteur.fr/fr/team/national-collection-of-cultures-of-microorganisms/.
