# Supplementary material for: Safety and functional enrichment of gut microbiome in healthy subjects consuming a multi-strain fermented milk product: a randomised controlled trial
Source: Sci Rep. 2020 Sep 29;10:15974. doi: 10.1038/s41598-020-72161-w (PMC7524715; doi:10.1038/s41598-020-72161-w)
Supplement: Supplementary file 1 — Supplementary Information [file 41598_2020_72161_MOESM1_ESM.docx]

**Safety and functional enrichment of gut microbiome in healthy subjects consuming a multi-strain fermented milk product: a randomised controlled trial**

Anne-Sophie Alvarez^1*^, Julien Tap^1*^, Isabelle Chambaud^1^, Stéphanie-Cools Portier^1^, Laurent Quinquis^1^, Pierre Bourlioux^2^, Philippe Marteau^3^, Eric Guillemard^1^_,_ Jürgen Schrezenmeir^4§^, Muriel Derrien^1§^

^*^ These authors contributed equally to this work

^§^ Shared last authors

1. Danone Nutricia Research, Palaiseau, France
2. Faculty of Pharmacy - Paris-Sud University, Chatenay-Malabry, France.
3. APHP. Sorbonne Université - INSERM-ERL 1157 - UMR7203, Hôpital Tenon, Paris, France
4. Clinical Research Center Kiel, Kiel Innovation and Technology Center, Schauenburgerstr. Kiel, Germany

**Corresponding author:** [muriel.derrien@danone.com](mailto:muriel.derrien@danone.com)

**Running title:** Functional contribution of probiotics to gut microbiota

**Supporting information**

**Supporting Material and Methods**

**Safety parameters**

Details of safety parameters measures are as follows: adverse events (number, occurrence, relationship to the study product, intensity, seriousness, action taken, subject outcome), physical examination (Vital signs: Systolic and diastolic blood pressure (SBP/DBP), heart rate (HR), body weight, body mass index, body temperature; clinical examination of gastrointestinal, cardiovascular, oto-rhino-laryngological, neurological, dermatological, musculoskeletal, urological/nephrological systems), blood analyses (hematology: hemoglobin, hematocrit, red blood cell count, white blood cell counts, platelets; metabolism profile: glucose, total cholesterol [TC], high density lipoprotein [HDL], low density lipoprotein [LDL], triglycerides [TG]; hepatic parameters: alanine aminotransferase [ALAT], aspartate aminotransferase [ASAT], gamma-glutamyltransferase [gGT]; kidney function: serum creatinin [CREAT]; inflammatory marker: C-reactive protein [hs-CRP]; thyroid marker: thyroid stimulating hormone [TSH]), calprotectin as inflammatory marker in feces, bowel habits (movements and feces consistency, based on Bristol Stool Scale (Lewis & Heaton 1997)), frequency of digestive symptoms (based on self-administered questionnaire including a 5 point-Likert scale on four items: abdominal pain/discomfort, abdominal bloating, flatulence [passage of gas], borborygmi/rumbling).

**Metagenomic Sequencing**

DNA was quantified by fluorimetry with the Qubit dsDNA HS Assay Kit (Thermo Fisher Scientific). We selected 107 samples from subjects who consumed three bottles a day (D0, D28 for both groups and additional D56 for Test 3) for analysis. Sequencing libraries were prepared with the Nextera XT DNA sample preparation kit, according to the manufacturer's instructions. (Nextera XT DNA Library Prep Reference Guide, Document # 15031942 v02 April2017, Illumina, San Diego, CA, USA). Library Profile and concentration were evaluated with the Agilent High Sensitivity DNA Kit in an Agilent 2100 Bioanalyzer. Libraries were pooled in sets of 14 to 16 samples and each pool was then run on a NextSeq500 sequencer (Illumina), in the 150 bp paired-end read configuration, with the NextSeq® 500/550 High Output Kit v2, in accordance with the manufacturer's instructions (NextSeq System Suite v2.1.2 2017). The PhiX Control library (v3) (Illumina) was combined with the amplicon library (expected at 1%). A mean of 35 million (± 8 million) paired-end reads were generated per sample.

**Read cleaning and filtering**

Raw data were processed to generate a gene count matrix with NGLess version 0.7 ^40^. A quality filter was first applied to the crude read data (fastq files). Reads with a quality < 20 and a length < 45 nucleotides were removed. An additional step was then performed to eliminate contamination with human sequences. Reads mapping onto the human genome hg19 sequence with at least 97% identity and a minimal match length of 45 nucleotides were discarded. We obtained 2,137,513,187 high-quality reads (mean of 19,976,759 reads per sample), which were used to build a gene reference catalog and to generate the count matrix (Supplementary Fig. S2).

**Augmented gene reference catalog construction**

The augmented catalog was built by enriching the Integrated Gene Catalog (IGC) ^41^ with the genes identified by the sequencing and *de novo* assembly of these 107 metagenomes and of the seven bacterial genomes present in the Test product (Supplementary Fig. S2). We reduced sequence redundancy and obtained a non-redundant augmented catalog of 10,081,514 million genes. These genes included 311,687 (3%) genes from this study: 307,910 genes for the 107 metagenomes and 3,777 genes for the seven bacterial genomes present in the Test product. These 311,687 genes included 209,815 new genes (2%) not present in the IGC catalogue and lacking IGC homologs at 95% identity and 90% coverage, and 101,872 genes (1%) that replaced shorter or more incomplete genes present in the IGC catalog.

The remaining reads were assembled *de novo* with ‘SPAdes’ version 3.10.0, with a *k-*mer value of 127 nucleotides, in the ‘meta’ mode. Genes were predicted with ‘MetaGeneMark’ 3.38 for ORF identification. We built a gene catalog representing all the predicted genes with no redundancy, for all the samples analyzed, by clustering genes on the basis of their similarity with the ‘CD-HIT’ program (threshold of 95% similarity). We used the Kyoto encyclopedia of genes and genomes (KEGG, http://www. genome.jp/kegg/) for the functional annotation of the catalog.

**Gene count matrix generation**

High-quality reads were mapped to the augmented gene catalog (Supplementary Fig. S2). Mapping and count matrix generation were also performed with NGLess. Reads not mapped onto the augmented gene catalog with a minimal identity of 95% and a minimal match length of 45 nucleotides were discarded. Shared read counts were weighted according to the number of unique mapped reads (NGLess option dist1). In brief, this type of counting involved two stages: First, the unique mapped reads (reads mapping to a single gene in the catalog) were attributed to the corresponding genes. Second, the shared reads (reads mapping with the same alignment score to multiple genes in the catalog) were attributed according to the ratio of their unique mapping counts. Finally, this gene count matrix obtained for all samples was normalized by gene length and scaled by sequencing depth. The scaled normalized gene abundance matrix of all samples was used as the starting point for the statistical analysis.

**Metagenomic species pan-genomes**

The relative abundance of metagenomic species pan-genomes (MSPs) was calculated by estimating the median for the top 50 genes for each core gene ^42^. We calculated relative abundances for 1661 MSPs from the Integrated Gene Catalog of 10 million genes ^42^. We then analyzed the correspondence of these MSPs with the augmented catalog built for this study. As the *L. rhamnosus* MSPs was not present among these 1661 MSPs, we constructed a pangenome for *L. rhamnosus* and integrated it into the catalog; it included 40 genomes of *L. rhamnosus* (14 genomes from an extensive metagenomics study ^61^, 25 genomes from Progenomes ^62^ and the *L. rhamnosus* CNCM I-3690 genome). For each of these 40 genomes, we filtered contigs over their entire length and selected contigs of more than 500 nucleotides. Genes were then predicted with Prodigal version 2.50 ^62^, according to the meta procedure. These genes were filtered over their entire length and genes of more than 100 nucleotides were retained. We also removed genes lacking one or both ends (3’ and 5’ codons). All the genes obtained from *L. rhamnosus* were then concatenated, and redundancy was eliminated with cd-hit, with the parameters cd-hit-est -aS 0.9 -c 0.95 -T 10 -M 0 -t 0 -d 100 -G 0.

Finally, we obtained 1662 MSPs, corresponding to 174 bacterial genera. Functional analysis was performed on GMM modules and gut brain modules ^63^ with KEGG gene annotations (omixerRpm version 0.3.0).

**Alpha diversity, beta diversity, qPCR**

We analyzed the qPCR data, MSPs richness (rarefied to 1700 counts) and gene counts (rarefied to 13.2 million mapped reads), and beta diversity (rarefied to 22,000 reads), in non-parametric statistical tests. For qPCR, we compared the Test 1 and Test 3 data at D14 and D28 (Mann-Whitney tests). For MSPs and gene counts, we first considered the difference between D28 and D0, and we performed statistical tests to compare Test 3 and Control 3 (Mann-Whitney tests). For beta-diversity, an intra-subject analysis was performed comparing D14, D28, and D56 with the corresponding baseline (D0), and an inter-subject (intra-dose) analysis was performed with the Kruskal-Wallis test. For all tests, alpha risk was set at 0.05 after FDR adjustment according to the Benjamini-Hochberg procedure.

We used the DESeq2 package (version 1.14.1, ^47^ to identify differentially abundant taxa (differences in genus count matrix (16S), MSPs count matrix (metagenomic) and metabolic pathway (gut module) between intervention groups and dose (Test, Control) across the study). The DESeq2 study model was set up to consider the composition of the microbiota at baseline (D0), while controlling for differences within individuals, as detailed in the DESeq2 tutorial. We included subjects with complete datasets for all time points (*N*=81 for 16S, (Test 1 (N=20), Test 3 (N=20), Control 1 (N=21), Control 3 (N=20)) and *N*=38 for metagenomics (Test 3 (N=18), Control 3 (N=20)). Significant fold-change differences between doses at each time point were evaluated with the negative binomial model-based Wald test implemented in DESeq2 (alpha risk set at 0.05 after FDR adjustment (default parameters)).

We used a zero-inflated beta regression model with random effects (ZIBR) (Chen & Li 2016) to evaluate the linear effect of dose, comparing the Test and Control groups over time (Control 1 and Control 3 grouped together, whereas doses were kept separate for the Test product). The model includes a logistic regression component, to model the presence/absence of a genus in the samples, and a beta regression component, to model non-zero microbial abundance, with each component including a random effect to account for the correlations between repeat measurements on the same subject. In the model, we assumed that consumption period (D14 and D28) would have a cumulative effect and that this effect would differ from that of non-consumption (D0 and D56), with a return to baseline values four weeks after the cessation of consumption. We therefore targeted genera displaying different linear modulations during the consumption period. This model complements and differs from the DESeq2 model, partly due to the way that ZIBR constrains the encoding of covariables and the testing of hypotheses. For both consumption and time effects, the alpha risk was set at 0.05 after FDR adjustment (default parameters). For complementary multivariate analysis, we applied a centered log ratio transformation (CLR) to genera selected by ZIBR (both consumption- and time-responsive), followed by PCA. Differences between groups (by dose) were assessed with the first two principal components, by Mann-Whitney analysis at each time point, followed by FDR adjustment according to the Benjamini-Hochberg procedure (applied within each parameter type).

**MSPs network**

MSPs were filtered on the basis of an abundance exceeding 1% in at least one subject, and were subjected to sparse inverse covariance estimation for ecological association and statistical inference (SPIEC-EASI) ^49^. SPIEC-EASI was used to construct a network between MSPs (D0 and D28) in the Test group, using default parameters. In this approach, the centered log ratio transformation normalized the data and the classical framework of Gaussian graphical models was then applied. This method uses pseudocounts to avoid zeros (Kurtz et al 2015). The network graphical representation was generated with igraph (version 1.2.4.1) and ggraph (version 2.0.0). Tidygraph (version 1.1.2) was used to compute the shortest (geodesic distance) path between all MSPs.

**Supporting Results**

***Subject enrolment, population at baseline and compliance***

In Table 1, subjects characteristics at baseline showed that approximately one-third of the global population were current smokers. The proportion of current smokers in the Test 1 group, was more than twice that in the Control 1 group (48 % versus 22 %). The number of cigarettes smoked per day, however, was similar between Test and Control groups (data not shown). Subjects reporting a regular physical activity varied between 35 and 75 % with a higher proportion in Test groups. Few subjects had at least a medical or a surgical history in each group (mostly infection and appendicitis) with some higher proportion in Control 1 and Test 3 groups but clinical examination resulted normal for all subjects at inclusion whatever the system examined. Around 40% of the subjects had a concomitant medication (mainly contraceptives) with a slightly higher proportion in the Test 1 as compared to Control 1 group, mainly due to a higher contraceptive use. For safety parameters at baseline (day 0), only the mean (SD) concentration of blood hs-CRP was higher and slightly above normal value (up to 47,6 mmol/L) in Test 1 as compared to Control 1 group (54,8 (102,2) vs 18,6 (15,9) mmol/L) whereas there was no significant difference between groups at higher dosage (19.4 (20.7) vs 25.8 (50.9) mmol/L in Test 3 and Control 3 group respectively). At the individual level, subjects with slight abnormal values, estimated clinically relevant by the investigator, were randomized which was due to an inconsistency in the definition of healthy status provided in the protocol and the one in the CRF used for subject inclusion. These were considered as minor deviations that were taken into account in the results interpretation.

Major deviations from the protocol were observed in seven randomized subjects (7%): 2 in Test 1, 1 in Control 1, 2 in Test 3, and 2 in Control 3 group. The reasons for deviation included non-compliance with the interval between planned visits, antibiotics intake or premature withdrawal. As there were less than 10% major deviations, only the FAS and not the Per Protocol population was analysed consistently with the Statistical Analysis Plan. Missing data were only observed from 0 to 3 subjects per group according to the parameter measured. The SMC considered the quality of the study conduct and the data as sufficient to allow the global analysis of the study results, as based on the observations of few major protocol deviations, well balanced or no interfering imbalanced subject characteristics at baseline between groups, a good compliance to the study product consumption and few missing data.

***Safety evaluation***

**Bowel movements, Digestive Symptoms and Vital signs**

Changes in weekly defecation frequency and stool consistency scores during the 4-week product consumption or the follow-up periods were minimal in all four groups. For the same periods, the composite score and the frequency of digestive symptoms (abdominal pain, bloating, flatulence and rumbling) were also similar between Test and Control groups and low symptoms frequencies were observed (from 0 to 3 times per week mainly) whatever group was considered. Composite score of digestive symptoms decreased from baseline similarly in both Test and Control groups. No clinically significant changes in any of the vital signs were reported in any of the four groups during either the consumption period or follow-up. For these parameters, absolute value of Cohen’s d coefficient was lower than 0.5 whatever the period, which is consistent with a similar pattern between Test and Control groups.

**Biological parameters**

For each biological parameter, changes from baseline (day 0) after 4-week product consumption (day 28) and associated Cohen’s d coefficient are provided in (Supplementary Table S4). For results interpretation all the individual data of subjects with at least one abnormal value during the whole study period and their evolution over time were also considered. Parameters with Cohen’s d outside the -0.5 to 0.5 range included leucocyte count (-0.61) and neutrophil counts (-0.90) for Test 1 versus Control 1 group and calprotectin (-0.82) for Test 3 versus Control 3 group. For both leukocytes and neutrophil counts, there was no significant difference in number of subjects with abnormal values between Test 1 and Control 1 groups during the product consumption period and only a very low Cohen’d was observed at higher product dose for each parameter. For calprotectin, a normal value at baseline with an evolution toward a possible or confirmed inflammatory disease at day14 or day 28 was observed in 4 (Test 1), 3 (Control 1), 2 (Test 3) and 5 (Control 3) subjects, with similar results in the follow-up period. Cohen’s d for calprotectin was otherwise low for Test 1 versus Control 1 group (0.17). CRP showed a low Cohen’s d coefficient whatever the product dose and a significant evolution toward an abnormal value at day 28 in 2 (Test 1), 2 (Control 1), 2 (Test 3) and 0 (Control 3) subjects. For parameters which exceeded the limits of normal range at baseline, intervention did not significantly affect these parameters. Individual data analysis globally showed that clinically relevant changes in laboratory parameters were rare with minimal and equivalent changes after 4 weeks of product consumption for all parameters in all groups. This was based on different observations: a no significant evolution from baseline, an identical or non-significantly different number of subjects with abnormal values between groups, a non-repeatable observation at higher product dose or a low Cohen’s d coefficient.

**Supplementary Figures**

**Figure S1.** Overview of the bioinformatics pipeline used in this study

**Figure S2.** Augmented gene reference catalogue construction

**Figure S3.** Beta-diversity assessed by different metrics. Within-subject variability (versus baseline) and between-subject (intra-group, at given time point) throughout the study

**Figure S4.** PCA of selected genera following CLR transformation. A) Selected bacterial genera loadings projected onto the two first principal components (PC). B) Samples projected onto the (PC2) facetted as function of Group, Doses and visits.

**Figure S5.** Average KEGG orthologs relative abundance as function of Test product genes contribution during consumption. Universal distributed genes were extracted from Ciccarelli et al ^43^. Dashed line indicates the threshold used to select significant functional contribution KO gene set.
