# Supplementary figures and images for: Safety and functional enrichment of gut microbiome in healthy subjects consuming a multi-strain fermented milk product: a randomised controlled trial
Source: Sci Rep. 2020 Sep 29;10:15974. doi: 10.1038/s41598-020-72161-w (PMC7524715; doi:10.1038/s41598-020-72161-w)

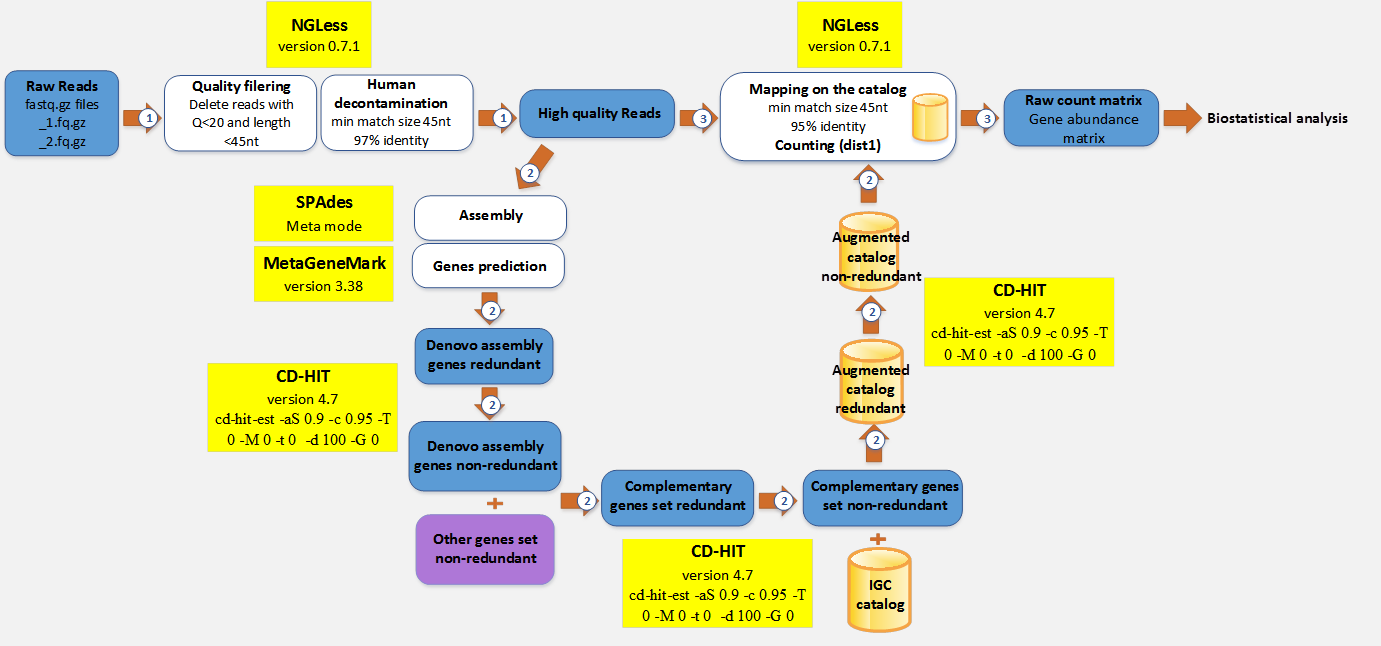

Supplement: Supplementary file 2 — Supplementary Figure S1. [file 41598_2020_72161_MOESM2_ESM.png]

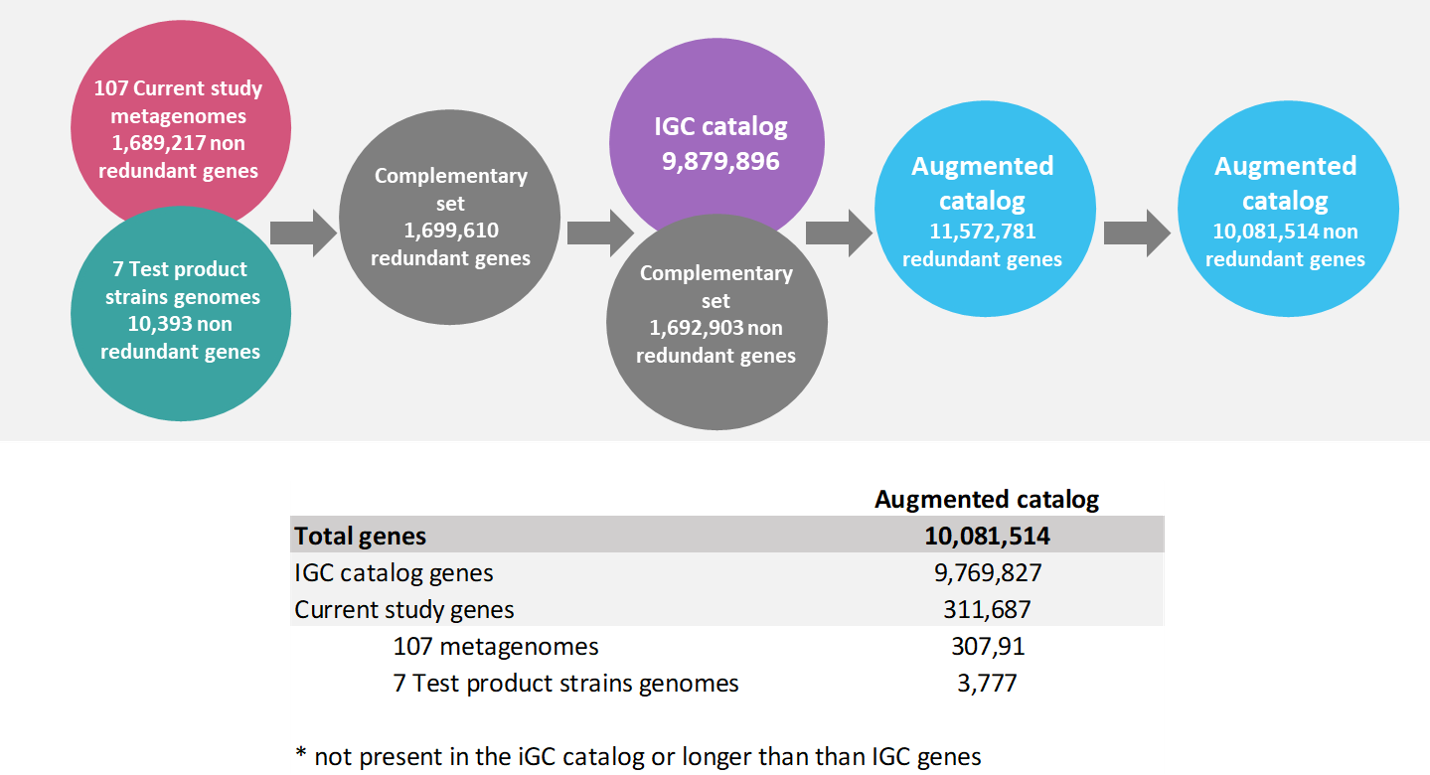

Supplement: Supplementary file 3 — Supplementary Figure S2. [file 41598_2020_72161_MOESM3_ESM.png]

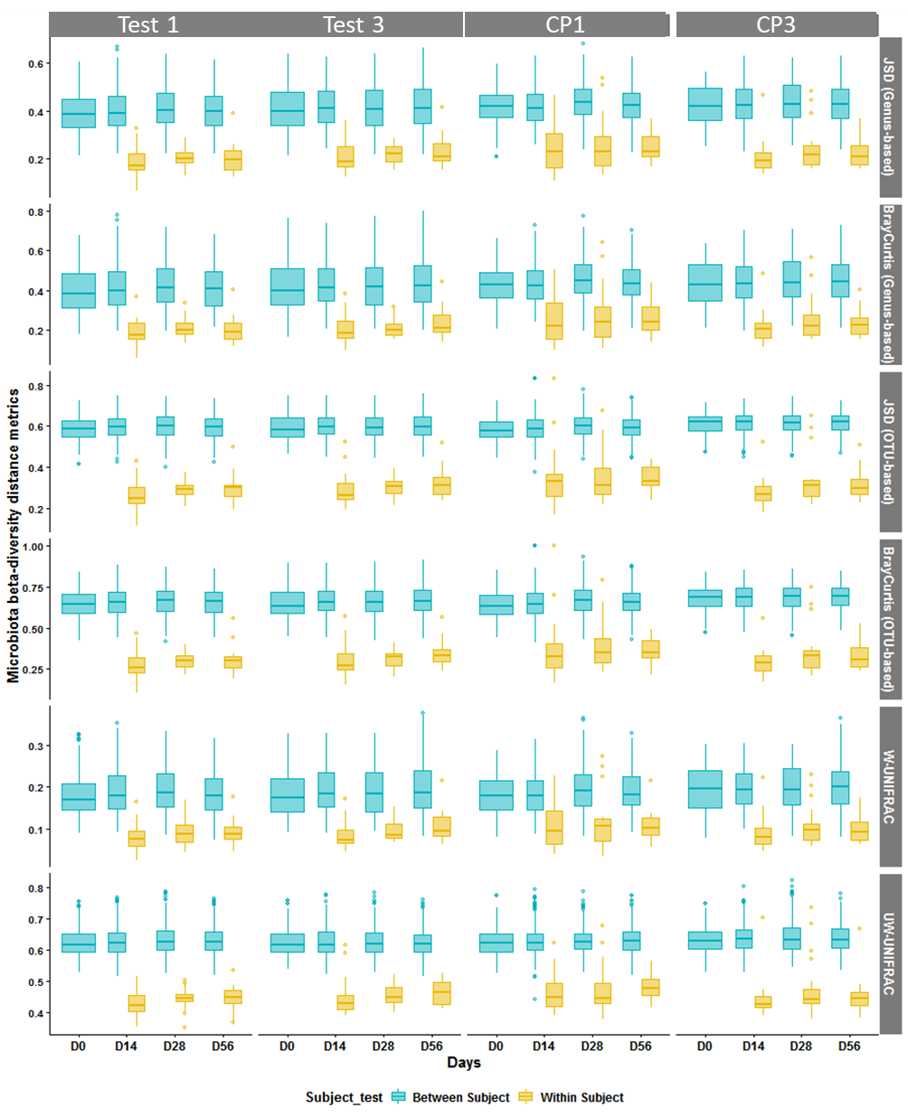

Supplement: Supplementary file 4 — Supplementary Figure S3. [file 41598_2020_72161_MOESM4_ESM.png]

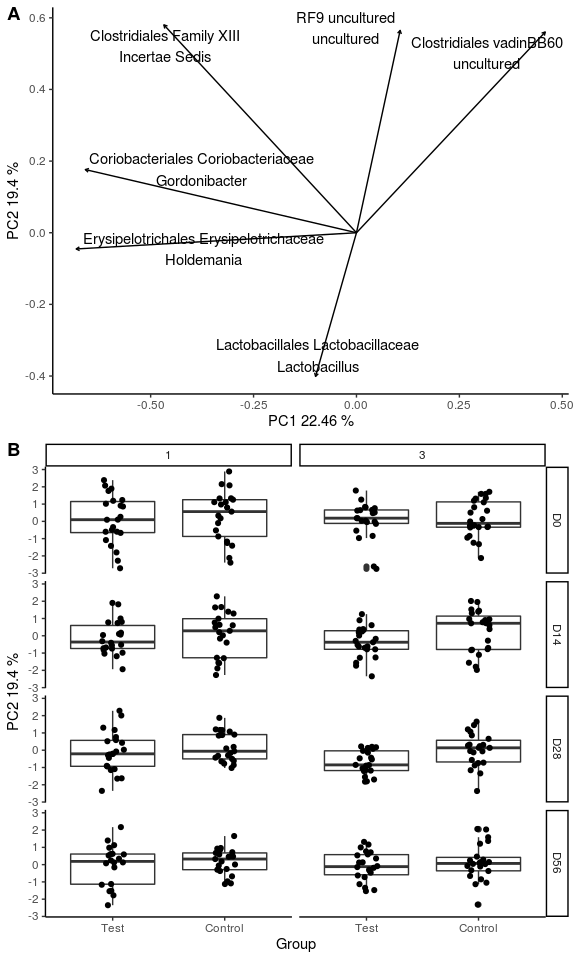

Supplement: Supplementary file 5 — Supplementary Figure S4. [file 41598_2020_72161_MOESM5_ESM.png]

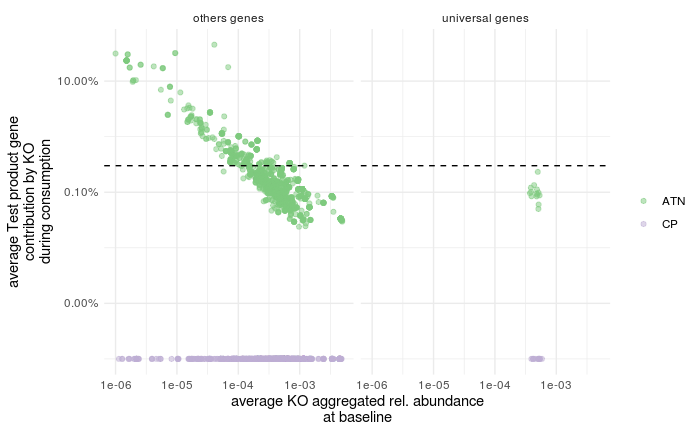

Supplement: Supplementary file 6 — Supplementary Figure S5. [file 41598_2020_72161_MOESM6_ESM.png]
