# Supplementary material for: Safety and functional enrichment of gut microbiome in healthy subjects consuming a multi-strain fermented milk product: a randomised controlled trial
Source: Sci Rep. 2020 Sep 29;10:15974. doi: 10.1038/s41598-020-72161-w (PMC7524715; doi:10.1038/s41598-020-72161-w)
Supplement: Supplementary file 7 — Supplementary Table S1. [file 41598_2020_72161_MOESM7_ESM.docx]

Table S1. Bacterial strain counts in Test product during the shelf life

| **Strains** | **Shelf life** | **Mean (Min-Max) (CFU/g)^1^** |
| --- | --- | --- |
| *Lactobacillus paracasei* CNCM I-1518 | Day 2 | 4.00.10^8^ (1.00.10^8^-1.00.10^9^) |
|  | Day 37 | 7.00.10^8^ (1.00.10^8^-1.00.10^9^) |
| *Lactobacillus paracasei* CNCM I-3689 | Day 2 | 3.40.10^8^ (1.00.10^7^-1.00.10^9^) |
|  | Day 37 | 3.70.10^8^ (1.00.10^7^-1.00.10^9^) |
| *Lactobacillus rhamnosus* CNCM I-3690 | Day 2 | 1.65.10^8^ (1.60.10^8^-1.70.10^8^) |
|  | Day 37 | 7.40.10^7^ (5.10.10^7^-9.70.10^7^) |
| *Streptococcus thermophilus* (CNCM I-2773, CNCM I-2835, CNCM I-2778) | Day 2 | 7.00.10^8^ (1.00.10^8^-1.00.10^9^) |
|  | Day 37 | 3.40.10^8^ (1.00.10^7^-1.00.10^9^) |
| *Lactobacillus bulgaricu*s CNCM I-2787 | Day 2 | 4.60.10^6^ (2.10.10^6^-7.10.10^6^) |
|  | Day 37 | 3.64.10^3^ (8.00.10^1^-7.20.10^3^) |

^1^ : Means were calculated as derived from measures carried out in each of the three batches of product used in the study. Due to exclusion of aberrant data, means were obtained from measures in two batches for *Lactobacillus rhamnosus* CNCM I-3690 and *Lactobacillus bulgaricus* CNCM I-2787 strains.
