# Supplementary material for: Safety and functional enrichment of gut microbiome in healthy subjects consuming a multi-strain fermented milk product: a randomised controlled trial
Source: Sci Rep. 2020 Sep 29;10:15974. doi: 10.1038/s41598-020-72161-w (PMC7524715; doi:10.1038/s41598-020-72161-w)
Supplement: Supplementary file 8 — Supplementary Table S2. [file 41598_2020_72161_MOESM8_ESM.docx]

**Table S2. Primers used in this study**

| **Target** | **Target gene** | **Amplicon size (bp)** | **Sequences (5’-3’)** | **References** |
| --- | --- | --- | --- | --- |
| *Lactobacillus paracasei* CNCM I-1518 | CRISPR | 281 | Forward : GTTAGCACCGCTTAAAGACG  Reverse :  GCCATAAGCGTGTTAGCCG | ^28^ |
| *Lactobacillus paracasei* CNCM I-3689 | cas gene | 112 | Forward  TTATCGATCCCAAGCTGGAC  Reverse :  ATCAGCGGTCCTTCAACATC | ^28^ |
| *Lactobacillus rhamnosus* CNCM I-3690 | putative transciptional regulator, Cro/CI family | 138 | Forward  GTGACAACCGCAATCACTTG  Reverse :  TATCGGTGCCATTGAGTGAA | This study |
