# Supplementary material for: Safety and functional enrichment of gut microbiome in healthy subjects consuming a multi-strain fermented milk product: a randomised controlled trial
Source: Sci Rep. 2020 Sep 29;10:15974. doi: 10.1038/s41598-020-72161-w (PMC7524715; doi:10.1038/s41598-020-72161-w)
Supplement: Supplementary file 10 — Supplementary Table S4. [file 41598_2020_72161_MOESM10_ESM.docx]

Table S4: Changes from baseline in biological parameters after 4-week product consumption (mean ± SD)

| **Parameter^a^** | **Test 1**  **(N=25)** | **Control 1**  **(N=23)** | **Cohen’s d** | **Test 3**  **(N=24)** | **Control 3**  **(N=24)** | **Cohen’s d** |
| --- | --- | --- | --- | --- | --- | --- |
| **Haematology** |  |  |  |  |  |  |
| Red Blood Cell count (10^12^/L) | -0.04 ± 0.15 | 0.09 ± 0.17 | -0.27 | -0.07 ± 0.19 | -0.08 ± 0.21 | 0.01 |
| Hematocrit (L/L) | 0.00 ± 0.02 | 0.01 ± 0.02 | -0.26 | 0.00 ± 0.02 | -0.01 ± 0.02 | 0.08 |
| Haemoglobin (mmol/L) | -0.14 ± 0.30 | 0.16 ± 0.30 | -0.32 | -0.18 ± 0.34 | -0.17 ± 0.38 | -0.02 |
| Platelet count (10^9^/L) | 2.08 ± 21.06 | 1.57 ± 24.98 | 0.01 | 2.50 ±24.92 | -2.39 ± 20.78 | 0.12 |
| Leucocyte count (10^9^/L) | -0.60 ± 1.49 | 0.35 ± 1.06 | -0.61 | -0.06 ± 0.90 | -0.18 ± 1.10 | 0.06 |
| Lymphocyte count (g/L) | -0.03 ± 0.35 | -0.08 ± 0.46 | 0.08 | 0.02 ± 0.46 | -0.03 ± 0.24 | 0.10 |
| Monocyte count (g/L) | -0.01 ± 0.11 | 0.02 ± 0.10 | -0.16 | 0.00 ± 0.02 | -0.01 ± 0.02 | 0.40 |
| Neutrophil count (g/L) | -0.57 ± 1.40 | 0.42 ± 0.69 | -0.90 | -0.14 ± 1.07 | -0.13 ± 1.04 | -0.01 |
| Basophil count (g/L) | 0.01 ± 0.08 | -0.02 ± 0.12 | -0.42 | 0.00 ± 0.01 | 0.00 ± 0.01 | 0.01 |
| Eosinophil count (g/L) | 0.29 ± 1.00 | -0.39 ± 1.27 | 0.20 | 0.23 ± 1.31 | -0.26 ± 1.29 | 0.14 |
| **Hepatic** |  |  |  |  |  |  |
| Alanine aminotransferase (IU/L) | 1.39 ± 7.52 | -1.91 ± 5.84 | 0.41 | -0.23 ± 6.68 | -2.09 ± 10.87 | 0.15 |
| Aspartate aminotransferase (IU/L) | 1.17 ± 4.57 | -1.09 ± 4.09 | 0.44 | 0.50 ± 4.22 | -1.48 ± 6.50 | 0.17 |
| Gamma glutamyltransferase (IU/L) | 0.26 ± 4.05 | -0.91 ± 4.66 | 0.11 | -1.77 ± 11.30 | -1.74 ± 6.84 | -0.00 |
| **Renal** |  |  |  |  |  |  |
| Serum creatinine (µmol/L) | -0.77 ± 7.01 | 1.92 ± 7.03 | -0.18 | -0.40 ± 7.46 | -0.77 ± 9.20 | 0.03 |
| **Metabolic** |  |  |  |  |  |  |
| Total cholesterol (mmol/L) | -0.11 ± 0.45 | 0.10 ± 0.37 | -0.24 | -0.30 ± 0.64 | -0.13 ± 0.57 | -0.15 |
| Low Density Lipoprotein (mmol/L) | -0.10 ± 0.31 | 0.11 ± 0.26 | -0.31 | -0.22 ± 0.48 | -0.08 ± 0.36 | -0.17 |
| High Density Lipoprotein (mmol/L) | -0.02 ± 0.16 | -0.01 ± 0.17 | -0.02 | -0.07 ± 0.16 | -0.06 ± 0.16 | -0.02 |
| Triglycerides (mmol/L) | 0.17 ± 0.61 | 0.00 ± 0.49 | 0.21 | -0.05 ± 0.42 | 0.14 ± 0.45 | -0.42 |
| Glucose (mmol/L) | -0.03 ± 0.27 | 0.06 ± 0.36 | -0.31 | -0.13 ± 0.28 | -0.08 ± 0.37 | -0.11 |
| **Inflammatory** |  |  |  |  |  |  |
| C-Reactive Protein (mmol/L) | -25.25 ± 96.77 | -1.66 ± 14.54 | -0.32 | 6.49 ± 19.79 | -10.35 ± 44.37 | 0.43 |
| Calprotectin (µg/g) | 7.54 ± 25.22 | -0.91 ± 118.85 | 0.17 | -4.18 ± 24.93 | 12.78 ± 27.11 | -0.82 |
| **Thyroidal** |  |  |  |  |  |  |
| Thyroid Stimulating Hormone (mU/L) | -0.14 ± 0.64 | -0.15 ± 0.63 | 0.01 | -0.13 ± 0.48 | -0.09 ± 0.74 | -0.06 |

^a^ : Parameters measured in blood samples except Calprotectin (Stool samples).
